# Anthracycline-induced cardiotoxicity: targeting high-density lipoproteins to limit the damage?

**DOI:** 10.1186/s12944-022-01694-y

**Published:** 2022-09-01

**Authors:** Carmelita Abrahams, Nicholas J. Woudberg, Sandrine Lecour

**Affiliations:** Cardioprotection Group, Cape Heart Institute and Hatter Institute for Cardiovascular Research in Africa, Department of Medicine, Faculty of Health Sciences, University of Cape Town, Cape Town, 7935 South Africa

**Keywords:** Cardio-oncology, Anthracycline, High-density lipoproteins, Cardiac toxicity

## Abstract

Doxorubicin (DOX) is an anthracycline antibiotic frequently used against a wide range of cancers, including breast cancer. Although the drug is effective as a treatment against cancer, many patients develop heart failure (HF) months to years following their last treatment with DOX. The challenge in preventing DOX-induced cardiotoxicity is that symptoms present after damage has already occurred in the myocardium. Therefore, early biomarkers to assess DOX-induced cardiotoxicity are urgently needed. A better understanding of the mechanisms involved in the toxicity is important as this may facilitate the development of novel early biomarkers or therapeutic approaches. In this review, we discuss the role of high-density lipoprotein (HDL) particles and its components as possible key players in the early development of DOX-induced cardiotoxicity. HDL particles exist in different subclasses which vary in composition and biological functionality. Multiple cardiovascular risk factors are associated with a change in HDL subclasses, resulting in modifications of their composition and physiological functions. There is growing evidence in the literature suggesting that cancer affects HDL subclasses and that healthy HDL particles enriched with sphingosine-1-phosphate (S1P) and apolipoprotein A1 (ApoA1) protect against DOX-induced cardiotoxicity*.* Here, we therefore discuss associations and relationships between HDL, DOX and cancer and discuss whether assessing HDL subclass/composition/function may be considered as a possible early biomarker to detect DOX-induced cardiotoxicity.

## Introduction

Noncommunicable diseases are the main cause of death worldwide, with cancer predicted to be the leading cause and an important deterrent in increasing life expectancy in all countries in the twenty-first century [1]. In 2020, breast cancer was the most prevalent cancer type diagnosed in females with more than 2 million newly reported cases worldwide and almost 700 000 mortalities [2].

Previously, the diagnosis of a cancer had a poor outcome for many patients. Fortunately, the discovery of chemotherapy has allowed patients to survive and live longer [3]. The anthracycline chemotherapy, doxorubicin (DOX), is classified by the Food and Drug Administration (FDA) as the most effective and frequently used chemotherapy and is among the World Health Organization’s (WHO) model list of essential medicines [4, 5]. Although it is an effective drug in killing cancer cells, many cancer survivors will develop cardiomyopathy or congestive heart failure several months to years following their last treatment with DOX [6, 7]. Patients present with symptoms of heart failure (HF) once the damage to the myocardium has already occurred, which makes early detection of DOX-induced cardiotoxicity challenging (see review [8]). Understanding the mechanisms and role players involved in the pathology of DOX-induced cardiotoxicity is therefore key to assist in the discovery of potential early biomarkers. Recent data suggest that high-density lipoprotein (HDL) particles and key components in HDL may serve as early players for protecting cardiomyocytes against DOX-induced cardiotoxicity [9].

Although the primary function of HDL particles is to facilitate the reverse cholesterol transport (RCT) by exporting cholesterol from the periphery back to the liver for degradation, their functionality is diverse and includes anti-oxidative, anti-inflammatory, anti-apoptotic and anti-thrombotic properties (see review [10]). Most importantly, HDL particles exist in different subclasses which differ in their composition of functional proteins and lipids, thus allowing for the wide range of biological activities (see review [11]). There is evidence that multiple cardiovascular risk factors such as obesity and diabetes are associated with a shift in the subclasses of HDL particles, favouring dysfunctional HDL particles [12, 13]. Here, we review evidence from the literature supporting the hypothesis that an alteration in circulating HDL subclasses, composition and functionality may be associated with DOX-induced cardiotoxicity in cancer patients, thus suggesting that assessing HDL subclass/composition or function may serve as possible early biomarkers to assess DOX-induced cardiotoxicity.

### Doxorubicin-induced cardiotoxicity

While DOX is widely used as a therapy for cancer, many patients suffer from cardiac dysfunction consecutive to the treatment, with a decline in left ventricular ejection fraction (LVEF) that will progress into HF [14]. The American Society of Echocardiography and the European Association of Cardiovascular Imaging define clinical cardiotoxicity with DOX as a 10-percentage point decrease in LVEF from baseline to a value below the lower limit of 53% [14]. Subclinical cardiac toxicity is diagnosed when LVEF is superior to 53% but global systolic myocardial longitudinal strain (GLS) is decreased by at least 15% compared to baseline and/or cardiac biomarkers such as troponin become positive during follow up [14].

A meta-analysis reported that after a median follow up of 9 years of 22 815 cancer patients of whom the majority was breast cancer patients, 17.9% developed subclinical cardiotoxicity, 6.3% developed clinical overt cardiotoxicity and 10.9% of patients had overall cardiac events [15]. Similarly, in 2625 cancer patients on anthracycline therapy, after a median follow up of 5.2 years, cardiotoxicity developed in 9% of the patients with the highest incidence observed in the first year after completion of therapy [7]. In a smaller study including 64 cancer patients on a treatment regimen that included DOX, 14 patients developed cardiotoxicity [16]. The latter was characterised by a significant reduction in left ventricular ejection fraction (LVEF) and changes in electrocardiogram parameters where shortening of the QRS and prolongation of the QTc interval were observed within 3 months of completion of treatment, resulting in a cumulative incidence rate of 21.9% [16]. In the CARDIOTOX registry that included 865 cancer patients of whom 84.5% received anthracycline therapy, 37.5% of the patients developed cardiotoxicity [6].

The main mechanism of antitumour activity of DOX is to target the deoxy-ribonucleic acid (DNA) (see review [17]). Once DOX enters the cell, it inserts itself between DNA base pairs, (DNA intercalation), preventing DNA replication and ribonucleic acid (RNA) transcription [18]. DOX also targets topoisomerase, which cuts DNA strands, allowing it to uncoil for DNA replication to occur. DOX stabilizes the conformation where the DNA strand is cut and bound to topoisomerase, thereby preventing DNA resealing. This will result in cellular senescence or apoptosis [4, 17].

The main mechanism of how DOX induces toxicity to healthy tissues is believed to be mediated by oxidative stress. However, in preclinical studies the use of anti-oxidants are not able to protect against DOX-induced cardiotoxicity doubting the role of generating reactive oxygen species (ROS) as the main mechanism for toxicity [19, 20]. Several other mechanisms are therefore also likely to occur, including mitochondrial dysfunction, inflammation, DNA damage, disruption of protein degradative pathways, signalling of cell death pathways and damage to cardiac progenitor cells.

#### Oxidative stress

Upon entering the cell, DOX undergoes redox cycling which results in the production of the free radical superoxide (O_2_^• ®^) and hydrogen peroxide (H_2_O_2_). Both enter the Harber-Weiss and Fenton reaction, catalysed by the presence of ferrous iron (Fe^2+^) to form hydroxyl radicals (^•^OH) (see reviews [21, 22]). The metabolite formed, doxorubicinol, further enhances oxidative stress by increasing free intracellular iron via a decrease of the iron scavenger protein, ferritin, and the inhibition of transferrin receptor breakdown thus resulting in more iron to enter the cell [23]. Nitric oxide synthase (NOS) also plays a key role in DOX-induced oxidative stress [24]. DOX exploits endothelial NOS (eNOS) to increase superoxide formation while decreasing nitric oxide (NO) production. It also targets NO directly to generate reactive nitrogen species [5, 25]. If these radicals are not removed, they accumulate and damage cellular membranes, organelles, DNA, and proteins by facilitating apoptosis [26]. The cell has an endogenous anti-oxidative defence system to neutralise ROS, however, cardiac tissue has reduced anti-oxidative enzymes compared to other tissues, thus making it a more vulnerable target [27]. DOX further decreases the endogenous anti-oxidative enzymes, glutathione and catalase leaving the heart highly susceptible to oxidative stress [25].

#### Mitochondrial dysfunction

The mitochondria are one of the major targets for DOX-induced cardiotoxicity and it involves several mechanisms of injury (see reviews [28, 29]). The mitochondria generate adenosine triphosphate (ATP) through mitochondrial respiration which involves the transfer of electrons in the electron transport chain (ETC) (see review [29]). Cardiomyocytes have a high load of mitochondria per unit volume to provide energy for its contractile function (see review [30]). DOX intercalates with mitochondrial DNA (mtDNA) and associates with mitochondrial inner membrane proteins, cardiolipin and complex I, preventing transcription and generating free radicals. The latter, oxidize proteins, lipids and nucleic acids leading to mitochondrial dysfunction and opening of the mitochondrial permeability transition pore (mPTP), thus resulting in apoptosis (see review [30]). ROS further promote accumulation of cytosolic calcium (Ca^2+^) by interacting with ryanodine receptors found on sarcoplasmic reticulum (SR) resulting in the release of Ca^2+^ into the cytosol. The mitochondria are located near SR within cardiomyocytes and aim to balance the cytosolic Ca^2+^ levels by retaining it. Accumulation of Ca^2+^ above the mitochondrial threshold, triggers opening of the mPTP, resulting in the loss of mitochondrial membrane permeability and ultimately the release of cytochrome c activating the intrinsic pathway of apoptosis [31]. As mentioned previously, DOX binds to cardiolipin which anchors mitochondrial membrane proteins such as cytochrome c [32]. However, cardiolipin has a high affinity to bind DOX making it no longer available to bind to cytochrome c contributing to the activation of the intrinsic pathway of apoptosis [32, 33]. DOX further disrupts mitochondrial ATP production through supressing genes coding for proteins part of the ETC, inactivating the complexes within the ETC and reducing the expression and activity of adenine nucleotide translocase (ANT) involved in ATP production [34–36]. DOX treatment disrupts iron homeostasis resulting in the accumulation of iron in cardiomyocytes, preferentially within the mitochondria [37, 38]. Iron contributes to ROS generation and hereby promotes mitochondrial injury (see reviews [21, 22]). The turnover of mitochondrial proteins in cardiomyocytes are 16–18 days. However, dysfunctional mitochondria persists 5–6 weeks after the last DOX treatment indicating that the mechanisms of cardiotoxicity effects successive generations of mitochondria and is not eliminated by the removal of defective mitochondria (see review [28]).

Maintaining mitochondrial function by preserving mitochondrial topoisomerase 1 (Top1mt) may offer protection against DOX-induced mitochondrial dysfunction. Top1mt maintained mtDNA stability and biochemical functions in wildtype mice on DOX treatment while Top1mt knockout mice were sensitive to DOX-induced mechanisms of injury [39].

#### Inflammation

Inflammation plays a key role in the development of HF with inflammatory cytokines contributing to progressive HF (see review [40]). DOX treatment results in the upregulation of pro-inflammatory cytokines, such as tumour necrosis factor alpha (TNF-α), interleukin-6 (IL-6) and a reduction of the anti-inflammatory cytokine interleukin-10 (IL-10). These early changes contribute to cardiac remodelling and the progression of late-onset cardiomyopathy [41]. Similarly, in a model of subacute DOX-induced cardiotoxicity, an increase in TNF-α, IL-6, interleukin-1β (IL-1β) is associated with impaired systolic function [42].

#### DNA damage

As mentioned previously, DOX induces cancer cell death by exploiting the activity of the topoisomerase enzyme [4, 17]. Topoisomerase exists in several isoforms, type I or II, which can be further subdivided into type α or β isoforms [23, 43]. In mammalian cells, type IIα are mainly found in proliferating cells such as cancer cells while type IIβ are mainly found in quiescent cells, such as cardiomyocytes [43]. In a knockout mouse model where the gene encoding for cardiomyocyte topoisomerase IIβ was deleted, cardiomyocytes were protected against DNA double strand breaks, defective mitochondrial biogenesis, and ROS formation. Furthermore, these mice were protected from progressive DOX-induced HF [44]. Dexrazone is an iron-chelator that protects against DOX-induced cardiotoxicity. Initially, it was understood that this drug protects by chelating free intracellular iron and hereby reduces ROS generation [45]. However, recent studies suggest that it protects against DOX-induced cardiotoxicity by degrading topoisomerase IIβ, hereby reducing the available pool of topoisomerase IIβ for DOX to bind and induce DNA damage [45, 46]. In the same model, the iron chelating metabolite ADR-925 was not able to protect against DOX-induced cardiotoxicity thus suggesting that topoisomerase II poisoning is a key role player in DOX-induced cardiotoxicity [45].

#### Protein degradative pathways

The ubiquitin proteasome system (UPS) and autophagy are protein degradative pathways which are active at basal conditions and during cellular stress to promote cell survival and development, however their dysregulation could lead to cell death (see review [47]). The UPS mediates the degradation of a wide range of cellular proteins and could also target specific proteins for degradation via ubiquitin E3 ligases [48]. In vitro and in vivo DOX treatment upregulated the E3 ligases, Atrogin-1 and muscle ring finger-1 (MuRF1) respectively, resulting in atrophy of cardiomyocytes [49, 50]. These findings also translate to the clinical setting where cancer patients undergoing chronic anthracycline treatment has a significant loss of cardiac mass as early as 1 month after initiation of treatment and continued throughout the 6 months follow up [49]. Autophagy degrades long lived proteins, protein aggregates and organelles (see review [47]). The role of autophagy in DOX-induced cardiotoxicity is unclear due to differential findings in experimental studies whether DOX upregulates or inhibits autophagy, and whether stimulating or inhibiting autophagy would protect against DOX-induced cardiotoxicity (see reviews [29, 51]). However, many experimental studies suggest that DOX induces cardiotoxicity through disrupting autophagy flux instead. It inhibits lysosomal degradation of the autophagosomes resulting in the accumulation of autophagosomes and autolysosomes within the cell and generating ROS [52, 53]. Lightening the load of autophagosomes for degradation by the lysosome, slowing autophagy initiation, and restoring lysosomal function restores autophagy and protects against DOX-induced cardiotoxicity [52, 53].

#### Metabolic alterations

DOX treatment is also associated with changes in the use of metabolites as an energy source, and the disruption of iron homeostasis within cardiomyocytes [54, 55]. Fatty acids are the main energy source of cardiomyocytes, however under stress and mitochondrial dysfunction they can switch to utilizing other substrates for energy production such as glucose. With DOX treatment, isolated cardiomyocytes have increased translocation of the glucose transporter 1 (GLUT1) with increased glucose uptake 1 h after treatment and returning to normal 3 h later [55]. Similarly, DOX treatment promoted increase in the oxidation of glucose, pyruvate, and lactate accompanied with the inhibition of oxidation of the long chain fatty acids in the cardiomyocytes of Sprague Dawley rats [54]. DOX treatment therefore promotes glucose oxidation and inhibits the oxidation of fatty acids. Glucose oxidation under pathological conditions may initially be helpful to meet the cellular energy demand, however in the long term it may not be sufficient and result in energetic failure instead (see reviews [29, 56]).

Accumulation of free intracellular iron contributes to the generation of ROS and consequently mitochondrial dysfunction as outlined above (see review [29, 57]). Regulatory mechanisms are in place to maintain iron homeostasis within the cell such as iron regulatory protein-1 (IRP-1). IRP-1 modulates the expression of proteins involved in the storage and transport of iron within the cells that includes the transferrin receptor and ferritin [58]. Transferrin receptor promotes the transport of iron into the cell, while ferritin stores iron intracellularly. The metabolite, doxorubicinol, irreversibly inactivates the activity of IRP-1 and IRP-2 [58]. It further increases intracellular iron by promoting the release of iron from ferritin, and upregulating the expression of transferrin receptor [59, 60]. DOX also disrupts the intracellular localization of iron by increasing the binding of intracellular iron to ferritin, however it reduces the release of iron from the mitochondria resulting in mitochondrial iron accumulation and consequently mitochondrial dysfunction and ferroptosis (see review [57]).

#### Cell death pathways

DOX-induced damage leads to cardiomyocyte death which may occur in a regulated or unregulated manner. Regulated cell death is under the control of biomolecules involved in signalling pathways while unregulated cell death occurs suddenly without the control of signalling pathways (see review [51]). Apoptosis is the most well characterized form of regulated cell death and is activated either by the intrinsic or extrinsic pathway. DOX-induced mitochondrial dysfunction, and upregulation of Bcl-2-associated X protein (Bax) results in the release of cytochrome c into the cytosol consequently activating the intrinsic pathway of apoptosis. Other mechanisms include the downregulation of anti-apoptotic proteins that includes GATA-binding protein 4 (GATA4) and B cell lymphoma extra-large (BcL-XL), and the inactivation of prosurvival pathways such as Phosphatidylinositol-3-Kinase/Protein Kinase B (PI3K/Akt) (see review [61]). Furthermore, DOX also upregulates proteins involved in the initiation of the extrinsic pathway of apoptosis, Fas/FasLand downregulates proteins involved in its inhibition such as FLICE-like inhibitory protein/ Fas-associated death domain-Like Interleukin-1β-Converting Enzyme (FLIP/FLICE) (see review [61]). Other forms of regulated cell death are also implicated in DOX-induced cardiotoxicity including ferroptosis which is activated by iron overload and accumulation of lipid peroxides, necroptosis activated by the release of TNF-α and pyroptosis stimulated by the increase in DOX-induced inflammation (see review [51]).

#### Cardiac progenitor cells

Endogenous cardiac progenitor cells (CPCs), c-kit positive and multipotent cells in the heart contribute to tissue repair and homeostasis in several diseases [62]. In an in vitro model of DOX-induced cardiotoxicity, human CPCs show activation of senescence and pro-apoptotic pathways [63]. Furthermore, human CPCs failed to restore structural or functional damage in the heart tissue of mice treated with DOX (see review [64]). The same effects are observed in the clinical setting in cancer patients treated with DOX. Those who died of HF presented with increased DNA damage and cellular senescence in their pool of CPCs compared to age matched controls who died of a non-cardiovascular cause. DOX depletion and damage to the pool of CPCs, may contribute to the high susceptibility of the heart to DOX-induced cardiotoxicity (see review [64]).

It is important to highlight that the severity of DOX-induced cardiotoxicity may be influenced by multiple factors including age and sex (see review [65]). Animal studies suggest that male adult rodents are more susceptible to DOX-induced cardiotoxicity (see review [65]. Unfortunately, data in juvenile animals are missing. Clinical studies suggest that the sex is a risk factor for DOX-induced cardiotoxicity with females being more at risk in paediatric cancer patients and males being more at risk in adult cancer patients (see review [65]). These data suggest a beneficial effect of female sex hormones and/or a detrimental effect of the male sex hormones in DOX-induced cardiotoxicity.

Although DOX is an effective and widely used chemotherapeutic agent, it is detrimental to the myocardium. Monitoring left ventricular function with the best possible imaging tool and myocardial deformation with speckle tracking to measure GLS during therapy is recommended to detect early changes in the myocardium [6]. Unfortunately, echocardiography 2-dimensional (2DE) is not sensitive enough to detect minor changes in the left ventricle and is not ideal for longitudinal measurements due to variations in measures with re-testing. Instead, 3-dimensional (3DE) echocardiography may provide more accurate measurements with an experienced user [6, 66]. A marker that could detect early signs of DOX-induced cardiotoxicity, may benefit the patient at risk. Classic cardiovascular disease (CVD) biomarkers such as cardiac troponin T (cTn T), I (cTn I) and the N-terminal-pro B Type natriuretic peptide (NT-proBNP) have been considered to identify patients at risk for cardiotoxicity (see review [67]). NT-proBNP is elevated in breast cancer patients 6 weeks after chemotherapy and associated with cardiotoxicity [68]. Similarly, in a different study, the same elevation was observed at 3, 6 and 12 months follow up prior to a decline in LVEF and could predict 1 year mortality [69]. In these studies, no significant changes in the cardiac troponins were observed [68, 69]. In contrast, a different study reported a positive change in cTn I that was associated with cardiotoxicity in breast cancer patients on anthracycline treatment after 3 months with no change in NT-proBNP [70]. These discrepancies question the use of these traditional biomarkers as a reliable tool to predict DOX-induced cardiotoxicity.

Interestingly, HDL particles are traditionally associated with cardiovascular health and preclinical research suggests that healthy HDL particles are protective against myocardial damage induced with DOX [9]. There is mounting evidence in the literature suggesting an association between HDL characteristics and the progression of DOX-induced cardiotoxicity.

### High-density lipoproteins and the cardiovascular system

Lipoproteins are particles responsible for the transport of dietary fats, including triglycerides, cholesterol and fatty acids within the blood [71]. There are four main different types of lipoproteins: chylomicrons, very low-density lipoproteins (VLDL), low-density lipoproteins (LDL) and HDL (see review [71]). As early as 1988, the Framingham heart study demonstrated an association between patients with low levels of HDL-cholesterol (HDL-c) and cardiovascular mortality [72]. Several large-scale clinical trials were successful to raise plasma HDL-c using cholesteryl ester transfer protein (CETP) inhibitors with an aim to decrease incidence of adverse cardiovascular events. However, the shift in lipid biomarkers did not have a significant effect in altering major CVD events [73–76]. In fact, treatment with these inhibitors may have resulted in a shift of HDL subclasses, leading to an alteration in the functionality of these HDL particles (see review [10]). Indeed, HDL particles exist in different subclasses which have different size and composition of proteins and lipids. The heterogeneity in the composition of HDL particles may alter its biological activity that is observed beyond its role to RCT.

#### High-density lipoproteins composition, subclass, and functionality

HDL particles are spherical shaped with an outer monolayer consisting of lipids and proteins surrounding an inside core of dietary lipids, that includes triglycerides and cholesterol esters. They are the smallest of the lipoproteins (5–12 nm) and have the highest density (1.063–1.21 g/ml) due to the high ratio of proteins to lipids [77, 78].

HDL particles are well known to be anti-atherogenic due to their RCT functionality [79]. It is largely mediated by apolipoprotein A1 (ApoA1) which makes up to 70% of all HDL proteins [77, 80]. ApoA1 binds to adenosine triphosphate binding cassette A1 (ABCA1), ABCG1 receptors and scavenger receptor class type B1 (SR-B1) on foam cells and peripheral tissue to collect cholesterol for metabolic degradation [79, 81]. HDL functionality further extends to include, although not limited to, anti-oxidative, anti-inflammatory, anti-thrombotic and anti-apoptotic activities.

The anti-oxidative function of HDL particles is mediated by multiple components present in the particles and these include the apolipoproteins, the lipid transfer proteins and different enzymes such as the paraoxonase (PON) [73], the CETP, the lecithin cholesterol acyltransferase (LCAT) and the platelet activating factor-acetylhydrolase (PAF-AH) (see review [82]). PON exists in 3 different isoforms and PON1 is exclusively associated with HDL [81, 83, 84]. PON1 neutralizes oxidative intermediates bound to oxidized LDL (oxLDL) and thus directly prevents the formation of atherosclerosis [85]. PON1 also protects endothelial and vascular smooth muscle cells in the arterial wall from protein modification and cellular toxicity preventing damage that will promote atherogenesis [86].

PAF-AH also contributes to the anti-inflammatory and anti-thrombotic activities of HDL particles. Whilst the majority of plasma PAF-AH is bound to LDL, only 20–30% is present in HDL particles [87]. Its protective effects were observed when human PAF-AH was directly administered to balloon injured carotid arteries in non-hyperlipidemic rabbits [88]. A reduction in the expression of inflammatory cytokine-induced expression of adhesion molecules was observed along with reduced macrophage infiltration and further less oxLDL was found accumulated in the area compared to the control [88]. Also, shear stress induced thrombosis was found to be reduced in the presence of PAF-AH [88]. In an in vivo model of ApoE ^−^/^−^ mice, PAF-AH was also found to display anti-atherogenic properties through anti-oxidative activity where it reduced endothelial cell adhesiveness and monocyte recruitment [89].

The sphingolipid, sphingosine-1-phosphate (S1P), also present in HDL particles, contributes significantly to the cardioprotective properties of HDL particles by promoting cell survival [9]. In models of ischemia/reperfusion (I/R) injury where the mechanism of injury includes oxidative stress and inflammation, S1P confers protection [9]. Most importantly, in the ex vivo model of I/R injury, reconstituted HDL enriched with S1P decreased infarct size significantly compared to the control receiving native HDL [90]. S1P preserved cell viability and mitochondrial integrity in H9C2 cells subjected to I/R injury by activating survival cell signalling pathways through activation of the signal transducer and activator of transcription 3 (STAT3) [91–93].

Multiple proteins and lipids have been identified as key components to contribute to the different physiological functions of HDL. Interestingly, the composition of these proteins and lipids found in HDL particles varies function to the type of HDL subclass (see review [10]).

The subclasses of HDL can be separated with ultracentrifugation based on their protein to lipid ratio, predominantly HDL2 and HDL3 (see review [10]). HDL2 is larger (8.8 nm -12.0 nm) and less dense (1.063 -1.125 g/mL), while HDL3 is smaller (7.2 – 8.8 nm) and denser (1.125–1.21 g/mL). These subclasses can further be divided into subpopulations according to their particle size in a descending order: HDL2b, HDL2a, HDL3c, HDL3b and HDL3a (see review [11]). The composition of these HDL subpopulations directly influences their biological functionality. HDL3 plays a more prominent role as an antioxidant with increased activity of the enzymes PON1 and PAF-AH (see reviews [11, 94]). It is also more resistant against oxidative modification compared to the larger, less dense HDL2 subpopulation (see reviews [11, 94]). In addition, HDL3 is more efficient in preventing in vitro copper induced lipid peroxide oxidation of LDL, compared to HDL2 [95]. Both HDL2 and HDL3 inhibit the cytokine, TNF-α induced vascular cell adhesion molecular-1 expression in endothelial cells in a concentration dependent manner [96]. However, HDL3 has a greater inhibitory effect compared to HDL2 [96]. Furthermore, HDL3, has greater capacity for RCT, thrombosis reduction and anti-apoptotic functions [97]. Interestingly, S1P associates more with HDL3 [94, 98]. It is therefore likely that the composition of HDL particles influences its cardioprotective properties. Note that care should be taken when isolating HDL as density ultracentrifugation can also co-isolate extracellular vesicles and this may negatively impact on the HDL particle analysis and conclusions of studies (see reviews [99, 100]).

Although scant information on sex differences is available in the literature, there seems to be a sex difference in HDL subclass distribution, with females having a higher value of large HDL subclass compared to men [101]. However, this difference is less with aging as a decrease in large HDL subclass is observed during the menopause transition in women [101, 102]. There is also growing evidence that, in disease states, HDL particles undergo modification, lose functional proteins and bioactive lipids resulting in a shift in their composition and causing them to lose their protective properties.

#### Changes in high-density lipoprotein subclasses, functionality and composition in pathophysiological conditions

There is strong evidence in the literature suggesting that a shift in HDL subclasses and functionality occurs in the presence of existing CVD and other conditions that increase the risk for developing CVD. In HF patients with reduced EF (HFrEF) or preserved EF (HFpEF), the small HDL subclass was decreased, and the large HDL subclass was increased compared to the healthy controls [103]. This effect was more pronounced in the HFrEF group than the HFpEF group. Furthermore, a reduction in the total HDL particles and the small HDL subclass was associated with an increased risk for an all-cause mortality [103]. The shift in HDL subclasses in these patients may further indicate that there is a change in HDL functionality as HDL from patients suffering from CVD were no longer protective [104, 105]. Indeed, HDL particles isolated from patients who have suffered from myocardial infarction were unable to protect the ex vivo heart against I/R injury, while the HDL isolated from healthy volunteers were protective [104]. Similarly, HDL from coronary artery disease patients failed to prevent oxidation of LDL in a cell free assay [105, 106]. In apparent contrast with these data, others did not observe any association between HDL subfractions and the presence or extent of coronary calcification [107].

The HDL particle profile is also altered in other pathophysiological conditions such as diabetes, obesity, hypertension and renal disease, which are known to be associated with an increased risk for cardiovascular related mortality [24, 108–110]. The hyperglycaemic, oxidative stress and low-grade inflammatory phenotype associated with diabetes further impaired HDL functionality by affecting the functional proteins associated with HDL [108, 111]. In the presence of inflammation, HDL loses its functional protein ApoA1 and associates with serum amyloid A (SAA). The association of SAA with HDL particles promotes atherogenesis by reducing the concentration of HDL-c, inhibiting the activity of LCAT and limiting HDL particles to prevent LDL oxidation [112]. S1P content is downregulated in both type 1 and type 2 diabetic patients [13, 113]. Furthermore, HDL isolated from diabetic patients are glycated causing S1P unable to protect cardiomyocytes against oxidative stress in vitro*,* while adding S1P to the diabetic HDL increased its S1P content and restored its cardioprotective capacity [13]. Hyperglycaemia further results in glycation of ApoA1, impairing its anti-atherogenic properties [108].

As ApoA1 is responsible for the maintenance of HDL structure and for controlling the activity of LCAT, alterations to the structure of ApoA1 consequently affects HDL [114–116]. ApoA1 has an amphipathic α-helix motif which unfolds and refolds to bind phospholipids and allows for the formation of discoidal HDL particles. Furthermore, as LCAT converts free cholesterol to cholesterol esters, ApoA1 adapts by bending and forming a scaffold structure to accommodate the size of the spherical HDL particle [117]. Glycation of ApoA1 impairs its ability to activate LCAT [115]. Depending on the degree of glycation and which amino acid residues are affected by glycation LCAT activity may either be enhanced or reduced [116]. The oxidative and inflammatory environment in atherosclerotic lesions results in the accumulation of oxidative cross-linked lipid poor ApoA1 with impaired functionality [118]. In these lesions, macrophages release myeloperoxidase which oxidises ApoA1 consequently preventing its binding to ABCA1 impairing cholesterol efflux [119]. Lastly, the oxidation of ApoA1 changes the structure of ApoA1 towards a more amyloidogenic protein promoting the deposition of amyloid fibrils [114].

Obesity and hypertension were associated with an increase in the smaller HDL subclass [109, 110, 120, 121]. Although the smaller subclass is suggested to have greater functionality due to its composition of functional proteins and lipids, its functionality may be compromised in these conditions [120, 121]. In obese hypertensive children, the small HDL subclass had reduced atheroprotective functionality, and CETP activity was more proatherogenic in the hypertensive patients compared to the normotensive patients [120]. Furthermore, the smaller HDL subclass was associated with a higher incident risk for incident hypertension while the large subclass was associated with reduced risk [109]. Furthermore, among patients with the metabolic syndrome smoking reduced plasma ApoA1 and a positive association with the HDL-c/ApoA1 ratio indicating a shift towards smaller HDL particle size [122]. However, HDL and ApoA1 functionality was not assessed in this study [122].

Surprisingly, HDL particles in end stage renal disease had greater cardioprotective functionality due to its increase in S1P concentration compared to healthy patients [123]. However, HDL functionality may be altered in kidney failure towards promoting inflammation and reduced anti-oxidative function due to a reduction in PON and glutathione peroxidase [24, 106].

Despite some discrepancies reported in the literature with regards to HDL subclass shifts associated with different pathophysiological conditions (that can, at least in part, be explained by the difference in the techniques used to measure these subclasses – see review [124]), it is evident that alterations in the inflammatory and oxidative environment in disease states alter the HDL subclass distribution and its composition, thus resulting in dysfunctional HDL. As mentioned previously, both cancer and DOX treatment are associated with conditions of inflammation and oxidative stress which are known to affect the composition and function of HDL. It is therefore likely that HDL particles may be negatively altered in cancer patients receiving DOX treatment.

### High-density lipoproteins and cancer patients

Many breast cancer patients present with an alteration in their lipid profile [125]. Cancer cells are highly proliferating and require cholesterol for the biogenesis of cell membranes, membrane rigidity and for cell signalling. The change in lipid profile is therefore mainly believed to be a consequence rather than a cause of the cancer (see review [126]). In breast cancer patients, the most frequent observation is lower or unchanged HDL-c levels (Table 1) together with higher total cholesterol and triglycerides compared to healthy patients. The apparent contradictions in changes in lipid profiles in cancer patients between studies, as seen in the Table 1, may in fact relate to the difference in age, the presence of other co-morbidities and lifestyle factors, the stage of cancer and the presence of therapies such as anthracyclines that are all known to alter the lipid profile [12, 127–130]. Very few studies report on the levels of HDL subfractions in cancer patients although lower HDL2 is consistently observed [131, 132]. Interestingly, breast cancer patients present with different histological characteristics, and those positive for the progesterone receptor were associated with the larger HDL subclasses while there didn’t seem to be any association between lipoprotein subfractions and the oestrogen receptor [133].

**Table 1:** Change in HDL-c and HDL subclass in breast cancer patients

Study	Patient enrolled	High Density Lipoproteins
[134]	*N* = 48 early-stage cancerous breast tumour patients *N* = 40 non-cancerous breast tumour patients	**↓** HDL-c in breast cancer patients vs. non-cancerous patients
[135]	*N* = 83 breast cancer patients (Stage I-IV) *N* = 83 healthy patients	**↓** HDL-c in Stage IV breast cancer patients vs. healthy patients
[136]	*N* = 58 breast cancer patients *N* = 105 healthy patients	No change in HDL-c vs. healthy patients
[132]	*N* = 30 breast cancer patients *N* = 30 healthy patients	**↓** HDL-c, HDL2 and HDL3 vs. healthy patients, an effect more pronounced for HDL-2 (-41%) than HDL-3 (-8%)
[131]	*N* = 56 postmenopausal breast cancer patients (Stage II-IV) *N* = 44 healthy patients	**↓** HDL-c, HDL2 and HDL3 vs. healthy patients Furthermore, advancement of disease affects lipid profile where, **↓** HDL-c in Stage IV breast cancer patients vs. Stage II breast cancer patients
[137]	*N* = 90 breast cancer patients *N* = 103 healthy patients	**↓** HDL-c in breast cancer patients vs. healthy patients
[138]	*N* = 100 breast cancer patients (Stage I-IV) *N* = 50 healthy patients	No change in HDL-c in breast cancer patients vs. healthy patients
[139]	*N* = 17 postmenopausal breast cancer patients *N* = 30 postmenopausal healthy patients	No change in HDL-c in breast cancer patients vs. healthy patients
[128]	*N* = 125 breast cancer patients (Stage I -IV) *N* = 70 healthy patients	**↓** HDL-c breast cancer patients vs. healthy patients
[140]	*N* = 100 pre- and postmenopausal breast cancer patients *N* = 100 healthy patients	No change in HDL-c in breast cancer patients vs. healthy patients
[127]	*N* = 120 breast cancer patients *N* = 60 healthy patient	No change in HDL-c vs. healthy patients
[141]	*N* = 60 pre- and postmenopausal breast cancer patients *N* = 60 healthy patients	No change in HDL-c vs. healthy patients
[142]	*N* = 249 early stage breast cancer patients *N* = 154 healthy patients	**↓** HDL-c in breast cancer patients ≤ 50 years old vs. healthy patients **↑** HDL-c in breast cancer patients ≥ 50 years old vs. healthy patients
[133]	*N* = 56 breast cancer patients (Stage I-II) No healthy patients included as controls	Large HDL subfractions associated with breast tumours expressing the progesterone receptor
[143]	*N* = 150 breast cancer patients *N* = 75 healthy patients	Unchanged HDL-c vs. healthy patients
[144]	*N* = 1054 breast cancer patients *N* = 2483 healthy patients	**↓** HDL-c in breast cancer patients < 60 years old vs. healthy patients Unchanged HDL-c in breast cancer patients > 60 years old vs. healthy patients

*HDL-c* High-density lipoprotein-cholesterol, *vs.* Versus

Mechanisms have been proposed on how cancer cells interact with HDL particles (for more details, see review [145]). In brief, cancer cells first accumulate cholesterol to enhance their proliferation. They have an increased expression of SR-B1, which stimulates the bi-directional transfer of cholesterol between plasma HDL particles and the cancer cells. It further reduces the expression of the ABCA1, which is key for the export of cholesterol from peripheral cells and cancer cells. Secondly, the presence of a tumour elicits an anti-tumour inflammatory response. The enhanced pro-inflammatory cytokine release alters plasma lipid levels, leading to a decrease and dysfunction of HDL particles as ApoA1 and PON1 activities are altered.

In addition, the interaction with cancer cells may not only decrease HDL-c levels but it affects its composition and functionality (see review [146]). The oxidative, inflammatory and dyslipidemic environment may negatively alter HDL composition by removing functional lipids and proteins components, leading to dysfunctional HDL (see review [146]). The enzymes, LCAT and PON, contribute to HDL’s antioxidant activity while LCAT also contributes to RCT function. The expression of LCAT and PON is lower in breast cancer patients and other cancer types along with a lower level of HDL-c. This suggests a reduced anti-oxidant defence during carcinogenesis and a reduced lipid transfer from tissues by HDL particles [147, 148]. These data support the few studies that report a shift in HDL subfractions in breast cancer patients [131–133].

A different aspect of the relationship between HDL and cancer, is the anti-tumour activity of ApoA1 [149]. In vitro, human ApoA1 reduced cell viability of ovarian cancer cells and prevented their invasion into the extracellular matrix. Furthermore, treatment with the ApoA1 mimetic increased sensitivity of ovarian cancer cells to the chemotherapy cisplatin [149]. Similarly, tumour bearing mice overexpressing ApoA1 had reduced tumour growth and metastasis of melanoma and lung cancer cells compared to mice lacking ApoA1 [150]. Subcutaneous injection of human ApoA1 to mice lacking ApoA1, prevented the formation and progression of tumours and reduced the size and growth of established tumours [150]. The anti-tumour effect of ApoA1 was achieved primarily through modulation of both the innate and adaptive immune response and reducing the expression and activity level of the extracellular matrix degrading enzyme matrix metalloproteinase-9 and the anti-apoptotic protein survivin within the tumour bed [122, 150]. The anti-tumour activity of ApoA1 against several cancer types and its ability to sensitize cancer cells to chemotherapeutic drugs highlights its potential as an adjuvant therapy for cancer patients.

### High-density lipoproteins and treatment with doxorubicin

In addition to the cancer itself, chemotherapy further affects plasma lipids [144, 151, 152]. Breast cancer patients on a treatment regimen that includes DOX present with lower HDL-c (see Table 2) and ApoA1 as well as higher LDL-cholesterol (LDL-c), triglycerides, and total cholesterol at the end of the treatment cycle compared to baseline [144, 152]. Interestingly, most patients with early stage breast cancer on anthracycline treatment have a decrease in plasma HDL-c during the course of treatment but this effect was restored 6 months following the last treatment [151]. The shift in lipid profile favouring proatherogenic lipoproteins could also be accompanied with increased markers for adiposity, insulin resistance, decrease in appendicular lean mass index and bone mineral density [153]. On the other hand, one study did not find a significant change in lipid metabolism in breast cancer patients receiving the anthracycline treatment, although patients had a dose dependent increase in fasting blood glucose level [154]. A lipid profile of low plasma HDL-c levels and high LDL-c levels are more frequently observed, with alterations in body composition, impaired glucose tolerance and insulin resistance may leave patients at a greater risk for CVD. To the best of our knowledge, no study has yet investigated an association between the change in HDL-c or the change in HDL subclass and functionality in cancer patients on DOX chemotherapy and their cardiovascular outcome. Furthermore, both breast cancer and DOX treatment alter the lipid profile, and it is currently unknown whether the one has a more pronounced effect on HDL function compared to the other one. Adequate animal models exploring the effect of breast cancer and/or DOX on lipid profile changes may assist in understanding the exact contribution of the cancer versus the drug on the shift of lipid profile observed in patients.

**Table 2:** Change in HDL-c in breast cancer patients on DOX treatment

Study	Sample	Chemotherapy regimen and duration	Results
[144]	*N* = 1054 breast cancer patients	TAC (*n* = 251) Cycled every 21 days for 6 cycles CEF (*n* = 9) Cycled every 21 days for 6 cycles AC-T (*n* = 134) AC cycled every 21 days for 6 cycles followed by T cycled every 21 days for 4 cycles	**↓** HDL-c prechemotherapy vs. post chemotherapy
[152]	*N* = 12 breast cancer patients	AC-P (*n* = 7) AC 3 times weekly for 4 weeks followed by P once weekly for 12 weeks CEF-T (*n* = 5) 3 times weekly for 3 weeks followed by T 3 times weekly for 3 weeks	**↓** HDL-c prechemotherapy vs. post chemotherapy

*A* Doxorubicin, *C* Cyclophosphamide, *E* Epirubicin, *F* 5-Flouracil, *T* Docetaxel, *P* Paclitaxel, *vs.* Versus

Considering that the cancer itself impacts on the plasma lipid levels and that cancer patients most often receive a combination of chemotherapeutic agents, exploring the effects of an individual agent on the lipid profile is not easy to pinpoint in the clinical setting. Additionally, the cancer itself could further have an impact on the plasma lipids. Sharma, Tuaine [152] investigated the effects of individual agents, including DOX, on lipoprotein metabolism in hepatocytes in vitro. The liver is responsible for metabolism and therefore receives and accumulates a high concentration of DOX [30]. DOX treatment directly targets the biogenesis of HDL particles by downregulating the expression levels of genes responsible for ABCA1 production and decreases ApoA1 protein levels [152]. HDL biogenesis starts with the release of ApoA1 from the liver into the circulation. It binds ABCA1 found in parenchymal cells, which transport phospholipids and unesterified cholesterol to ApoA1 to form nascent pre-β HDL-c [155]. DOX reduces expression of HMG-CoA reductase (HMGCR), a rate determining enzyme of cholesterol formation in the cell [152]. DOX, as an individual agent, decreases plasma HDL-c through inhibiting ApoA1 and cholesterol efflux, but the functionality and subclass of HDL particles was not investigated.

**Fig. 1:**
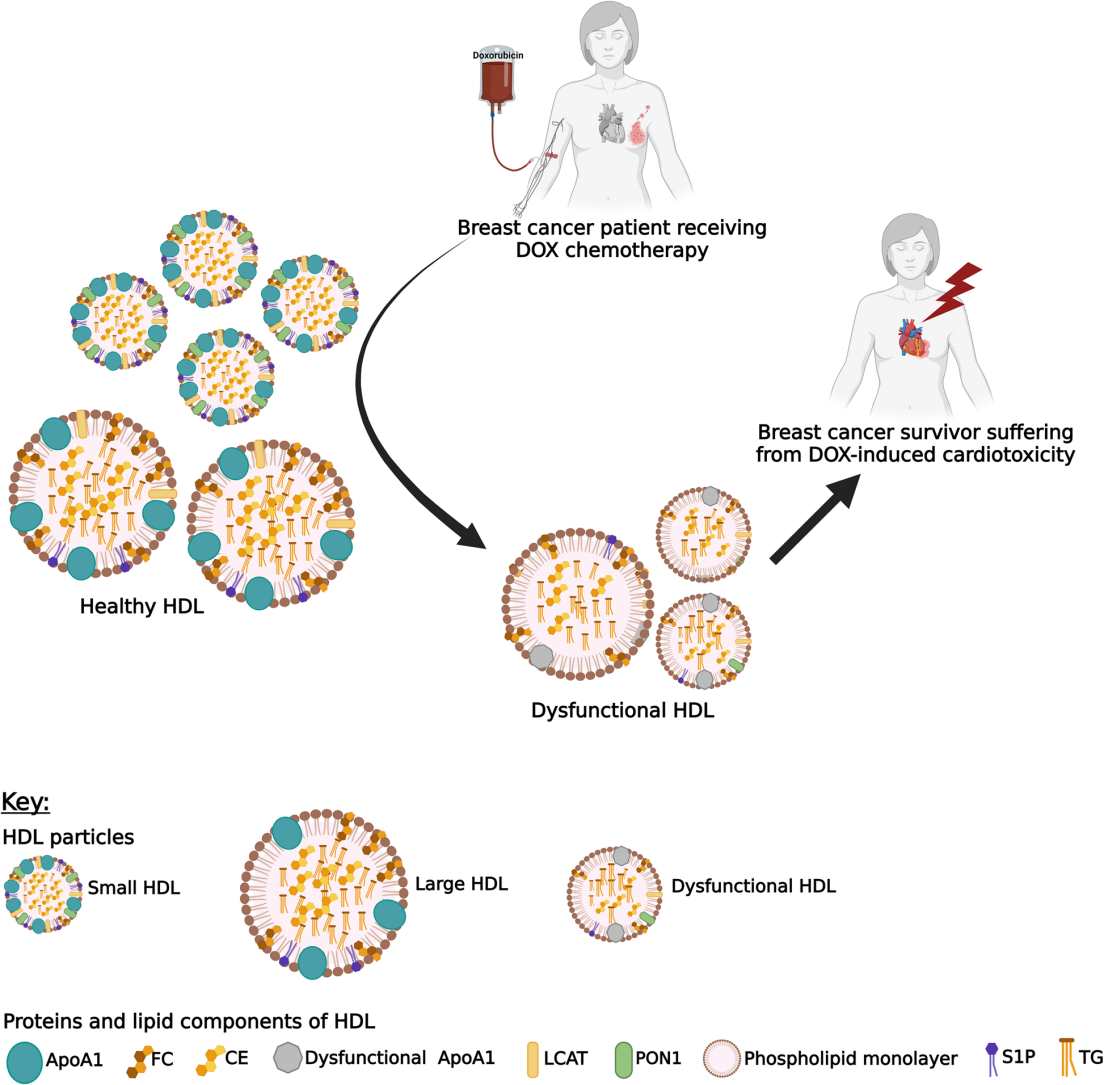
Proposed mechanism depicting the role of HDL in doxorubicin-induced cardiac toxicity. We propose that a shift in high-density lipoproteins (HDL) subclasses in breast cancer patients treated with doxorubicin leads to dysfunctional HDL with reduced anti-oxidative, anti-inflammatory, reverse cholesterol transport function and anti-apoptotic function that may facilitate the cardiac damage associated with the treatment of doxorubicin Abbreviations: *ApoA1* Apolipoprotein A1, *CE* Cholesteryl ester, *DOX* Doxorubicin, *FC* Free cholesterol, *HDL* High-density lipoprotein, *LCAT* Lecithin cholesterol acyltransferase, *PON1* Paraoxonase 1, *S1P* Sphingosine-1-phosphate, *TG* Triglyceride

We speculate that the mechanisms involved in DOX-induced cardiotoxicity could involve the formation of dysfunctional HDL that become unable to protect cardiomyocytes against the toxic effect of DOX. As treatment with DOX will result in ROS generation, disruption of intracellular iron homeostasis and mitochondrial dysfunction which all promote oxidative stress and inflammation. Dysfunctional HDL will lose their anti-inflammatory and antioxidant functions as characterized by a reduction in PON1 anti-oxidative functionality, structural and functional changes of its composition such as ApoA1 and S1P (see Fig. 1) [108, 111, 112, 114–116]. Although HDL-c levels are restored 6 months following DOX therapy, the return to physiological HDL subclass, composition and function has not been investigated. It is therefore unknown if HDL particles may contribute to the long term effects of DOX-induced cardiotoxicity.

There is however, preclinical evidence suggesting that certain HDL subclasses and specific HDL constituents such as S1P and ApoA1, have a positive outcome on DOX-induced cardiotoxicity [9]. Both HDL isolated from healthy volunteers (native HDL), and free S1P protect ventricular cardiomyocytes against DOX-induced cardiotoxicity in a dose dependent manner [9]. Further investigation into the specific role of HDL and its constituents, S1P and ApoA1, suggested that reconstituted HDL (rHDL) enriched with S1P decreased DOX-induced cell death, rHDL with S1P and ApoA1 had a stronger inhibitory effect while rHDL with ApoA1 alone did not offer protection [9]. S1P was found to counteract DOX-induced cell death through binding to S1P receptor 2, activating extracellular signal regulated kinase 1/2 and STAT3 [9, 156]. Based on the composition of functional proteins and lipids, the HDL subfractions may have different functions in protecting against DOX-induced cardiotoxicity. Interestingly, Durham, Chathely [157] found that ApoA1 overexpression in vivo limited the effects of DOX-induced cardiotoxicity, through protecting against cardiomyocyte atrophy, apoptosis and preserving cardiac function [157]. These effects were mediated by the activation of Akt in cardiac tissue. Akt promotes cell survival by protein synthesis, promoting hypertrophy and regulating the intrinsic pathway of apoptosis. Further investigation revealed, that the protective effects of ApoA1 were mediated through ApoA1 binding to SR-B1 in cardiomyocyte, as the protective effects were lost in mice lacking SR-B1 [157]. Also, investigations into the role of SR-B1 revealed that mice deficient in SR-B1 with endogenously expressed ApoA1, had higher susceptibility to DOX-induced cardiotoxicity compared to wildtype mice and ApoA1 treatment was not able to offer protection [157]. Interestingly, their HDL particles were not able to protect wildtype cardiomyocytes against DOX-induced apoptosis in vitro*.* On the other hand, HDL particles isolated from wildtype mice were not able to protect cardiomyocytes isolated from SR-B1 deficient mice against DOX-induced apoptosis [158]. These findings suggest the potential of ApoA1 as a therapeutic intervention against DOX-induced cardiotoxicity and that its protective effects are mediated through binding to SR-B1.

There are several risks for increased susceptibility to DOX-induced cardiotoxicity including the method of drug administration (see review [159]). The use of drug delivery vehicles for the administration of chemotherapeutic drugs, such as pegylated liposomal DOX has shown potential to eradicate the tumour while limiting toxicity compared to free DOX (see review [160]). Interestingly, HDL particles are suitable for drug delivery due to their structure allowing it to be loaded with drugs and presenting the advantage to be endogenous, to be degraded in vivo and to have a long half-life [161]. Loading DOX into rHDL with ApoA1, decreased cell viability of hepatocellular carcinoma cells with a greater concentration of DOX accumulated intracellularly compared to treatment with free DOX. This effect was weakened when the SR-B1 receptor was blocked, thus highlighting the fact that the increased efficacy of DOX involved the binding of ApoA1 to SR-B1 receptor. In vivo, there was a greater accumulation of DOX in the liver compared to the heart and other organs [161]. Most importantly, using rHDL particles as drug delivery vehicle did not compromise the antineoplastic activity of DOX in vivo as a greater decrease in tumour size and weight was found compared to the administration of DOX on its own [162].

## Conclusion

In conclusion, both the cancer cells and chemotherapy alter the plasma lipid profile in breast cancer patients with a decrease in HDL-c commonly observed. Few studies have explored HDL subfractions and HDL composition and all of them converge to the conclusion that the cancer environment leads to a shift in HDL subclasses and a change in HDL composition favouring dysfunctional HDL particles. Preclinical studies suggest that healthy HDL and rHDL enriched with S1P may protect against DOX-induced cardiotoxicity. Of note, most preclinical studies have been conducted in the absence of the cancer environment which may affect the HDL particle dynamic. In addition, it is important to take into consideration that multiple confounders including age, sex, ethnicity and multiple cardiovascular risk factors (i.e. diabetes, hypertension) may also influence the change in HDL subclass, functionality, and composition in cancer patients [121, 163–165]. Based on the above findings however, it is possible to speculate that an alteration in HDL particles, in terms of their composition and functionality, in cancer patients treated with DOX may contribute to the pathogenesis of DOX-induced cardiotoxicity. Further investigation into changes of HDL subclasses/composition/function with the progression of DOX-induced cardiotoxicity in a cancer environment could potentially identify key components that may serve as early biomarkers or may protect against DOX-induced cardiotoxicity.

## Data Availability

Not applicable.

## Abbreviations

OH: Hydroxyl radicals
2DE: 2-Dimensional echocardiography
3DE: 3-Dimensional echocardiography
ABCA1: Adenosine triphosphate binding cassette A1
ABCG1: Adenosine triphosphate binding cassette G1
ANT: Adenine nucleotide translocase
ApoA1: Apolipoprotein A1
ATP: Adenosine triphosphate
Bax: Bcl-2-associated X protein
BcL-XL: B cell lymphoma extra-large
Ca^2+^: Calcium
CETP: Cholesteryl ester transfer protein
CPCs: Cardiac progenitor cells
cTnI: Cardiac troponin I
cTnT: Cardiac troponin T
CVD: Cardiovascular disease
DNA: Deoxy-ribonucleic acid
DOX: Doxorubicin
eNOS: Endothelial nitric oxide synthase
ETC: Electron transport chain
FDA: Food and Drug Administration
Fe^2+^: Ferrous iron
FLIP/FLICE: FLICE-like inhibitory protein/ Fas-associated death domain-Like Interleukin-1β-Converting Enzyme
GATA4: GATA-binding protein 4
GLS: Global systolic myocardial longitudinal strain
GLUT1: Glucose transporter 1
H_2_O_2_: Hydrogen peroxide
HDL: High-density lipoprotein
HDL-c: High-density lipoprotein-cholesterol
HF: Heart failure
HFpEF: Heart failure preserved ejection fraction
HFrEF: Heart failure reduced ejection fraction
HMGCR: HMG-CoA reductase
I/R: Ischemia/reperfusion
IL-10: Interleukin-10
IL-1β: Interleukin-1β
IL-6: Interleukin-6
IRP-1: Iron regulatory protein-1
IRP-2: Iron regulatory protein-2
LCAT: Lecithin cholesterol acyltransferase
LDL: Low-density lipoprotein
LDL-c: Low-density lipoprotein-cholesterol
LVEF: Left ventricular ejection fraction
mPTP: Mitochondrial permeability transition pore
mtDNA: Mitochondrial deoxy-ribonucleic acid
MuRF-1: Muscle ring finger-1
NO: Nitric oxide (NO)
NOS: Nitric oxide synthase
NT-proBNP: N-terminal-pro B Type natriuretic peptide
O_2_^• ®^: Superoxide
oxLDL: Oxidized LDL
PAF-AH: Platelet activating factor-acetylhydrolase
PI3K/Akt: Phosphatidylinositol-3-Kinase/Protein Kinase B
PON: Paraoxonase
RCT: Reverse cholesterol transport
rHDL: Reconstituted high-density lipoprotein
RNA: Ribonucleic acid
ROS: Reactive oxygen species
S1P: Sphingosine-1-phosphate
SAA: Serum amyloid A
SR: Sarcoplasmic reticulum
SR-B1: Scavenger receptor class type B1
STAT3: Signal transducer and activator of transcription 3
TNF-α: Tumour necrosis factor-alpha
Top1mt: Mitochondrial topoisomerase 1
UPS: Ubiquitin proteasome system
VLDL: Very low-density lipoprotein
WHO: World Health Organization

## References

[1] Bray F, Ferlay J, Soerjomataram I, Siegel RL, Torre LA, Jemal A (2018). Global cancer statistics 2018: GLOBOCAN estimates of incidence and mortality worldwide for 36 cancers in 185 countries. CA Cancer J Clin.

[2] Global Cancer Observatory: Cancer Today. International Agency for Research on Cancer. Lyon, France. 2020. https://gco.iarc.fr/today. Accessed 27 Jul 2021.

[3] Curry H, Parkes S, Powell J, Mann J (2006). Caring for survivors of childhood cancers: the size of the problem. Eur J Cancer.

[4] Corremans R, Adão R, De Keulenaer GW, Leite-Moreira F, Brás-Silva C (2019). Update on pathophysiology and preventive strategies of anthracycline-induced cardiotoxicity. Clin Exp Pharmacol Physiol.

[5] Tacar O, Sriamornsak P, Dass CR (2013). Doxorubicin: an update on anticancer molecular action, toxicity and novel drug delivery systems. J Pharm Pharmacol.

[6] López-Sendón J, Álvarez-Ortega C, Zamora Auñon P, Buño Soto A, Lyon AR, Farmakis D (2020). Classification, prevalence, and outcomes of anticancer therapy-induced cardiotoxicity: the CARDIOTOX registry. Eur Heart J.

[7] Cardinale D, Colombo A, Bacchiani G, Tedeschi I, Meroni CA, Veglia F (2015). Early detection of anthracycline cardiotoxicity and improvement with heart failure therapy. Circulation.

[8] Shah KS, Yang EH, Maisel AS, Fonarow GC (2017). The role of biomarkers in detection of cardio-toxicity. Curr Oncol Rep.

[9] Frias MA, Lang U, Gerber-Wicht C, James RW (2010). Native and reconstituted HDL protect cardiomyocytes from doxorubicin-induced apoptosis. Cardiovasc Res.

[10] Woudberg NJ, Pedretti S, Lecour S, Schulz R, Vuilleumier N, James RW (2018). Pharmacological intervention to modulate HDL: what do we target?. Front Pharmacol.

[11] Camont L, Chapman MJ, Kontush A (2011). Biological activities of HDL subpopulations and their relevance to cardiovascular disease. Trends Mol Med.

[12] Woudberg NJ, Lecour S, Goedecke JH (2019). HDL Subclass Distribution Shifts with Increasing Central Adiposity. J Obes.

[13] Brinck JW, Thomas A, Lauer E, Jornayvaz FR, Brulhart-Meynet MC, Prost JC (2016). Diabetes Mellitus Is Associated With Reduced High-Density Lipoprotein Sphingosine-1-Phosphate Content and Impaired High-Density Lipoprotein Cardiac Cell Protection. Arterioscler Thromb Vasc Biol.

[14] Plana JC, Galderisi M, Barac A, Ewer MS, Ky B, Scherrer-Crosbie M (2014). Expert consensus for multimodality imaging evaluation of adult patients during and after cancer therapy: a report from the American Society of Echocardiography and the European Association of Cardiovascular Imaging. J Am Soc Echocardiogr.

[15] Lotrionte M, Biondi-Zoccai G, Abbate A, Lanzetta G, D’Ascenzo F, Malavasi V (2013). Review and meta-analysis of incidence and clinical predictors of anthracycline cardiotoxicity. Am J Cardiol.

[16] Kibudde S, Mondo CK, Kibirige D, Walusansa V, Orem J (2019). Anthracycline induced cardiotoxicity in adult cancer patients: a prospective cohort study from a specialized oncology treatment centre in Uganda. Afr Health Sci.

[17] Minotti G, Menna P, Salvatorelli E, Cairo G, Gianni L (2004). Anthracyclines: molecular advances and pharmacologic developments in antitumor activity and cardiotoxicity. Pharmacol Rev.

[18] Cummings J, Anderson L, Willmott N, Smyth JF (1991). The molecular pharmacology of doxorubicin in vivo. Eur J Cancer Clin Oncol.

[19] Breed JG, Zimmerman AN, Dormans JA, Pinedo HM (1980). Failure of the antioxidant vitamin E to protect against adriamycin-induced cardiotoxicity in the rabbit. Cancer Res.

[20] Berthiaume J, Oliveira P, Fariss M, Wallace KB (2005). Dietary vitamin E decreases doxorubicin-induced oxidative stress without preventing mitochondrial dysfunction. Cardiovasc Toxicol.

[21] Šimůnek T, Štěrba M, Popelová O, Adamcová M, Hrdina R, Geršl V (2009). Anthracycline-induced cardiotoxicity: overview of studies examining the roles of oxidative stress and free cellular iron. Pharmacol Rep.

[22] Hrdina R, Gersl V, Klimtová I, Simunek T, Machackova J, Adamcová M (2000). Anthracycline-induced cardiotoxicity. Acta Med.

[23] Mitry MA, Edwards JG (2016). Doxorubicin induced heart failure: Phenotype and molecular mechanisms. IJC Heart Vasc.

[24] Moradi H, Pahl MV, Elahimehr R, Vaziri ND (2009). Impaired antioxidant activity of high-density lipoprotein in chronic kidney disease. Transl Res.

[25] Songbo M, Lang H, Xinyong C, Bin X, Ping Z, Liang S (2019). Oxidative stress injury in doxorubicin-induced cardiotoxicity. Toxicol Lett.

[26] Octavia Y, Tocchetti CG, Gabrielson KL, Janssens S, Crijns HJ, Moens AL (2012). Doxorubicin-induced cardiomyopathy: from molecular mechanisms to therapeutic strategies. J Mol Cell Cardiol.

[27] Odom AL, Hatwig CA, Stanley JS, Benson AM (1992). Biochemical determinants of adriamycin® toxicity in mouse liver, heart and intestine. Biochem Pharmacol.

[28] Wallace KB, Sardão VA, Oliveira PJ (2020). Mitochondrial determinants of doxorubicin-induced cardiomyopathy. Circ Res.

[29] Huang J, Wu R, Chen L, Yang Z, Yan D, Li M (2022). Understanding Anthracycline Cardiotoxicity From Mitochondrial Aspect. Front Pharmacol.

[30] Carvalho C, Santos RX, Cardoso S, Correia S, Oliveira PJ, Santos MS (2009). Doxorubicin: the good, the bad and the ugly effect. Curr Med Chem.

[31] Zhang K, Kaufman RJ (2008). From endoplasmic-reticulum stress to the inflammatory response. Nature.

[32] Nicolay K, Timmers RJ, Spoelstra E, Van Der Neut R, Fok JJ, Huigen YM (1984). The interaction of adriamycin with cardiolipin in model and rat liver mitochondrial membranes. Biochim Biophys Acta Biomembr.

[33] Goormaghtigh E, Brasseur R, Ruysschaert J-M (1982). Adriamycin inactivates cytochrome c oxidase by exclusion of the enzyme from its cardiolipin essential environment. Biochem Biophys Res Commun.

[34] Oliveira PJ, Wallace KB (2006). Depletion of adenine nucleotide translocator protein in heart mitochondria from doxorubicin-treated rats—relevance for mitochondrial dysfunction. Toxicology.

[35] Marcillat O, Zhang Y, Davies KJ (1989). Oxidative and non-oxidative mechanisms in the inactivation of cardiac mitochondrial electron transport chain components by doxorubicin. Biochem J.

[36] Pointon AV, Walker TM, Phillips KM, Luo J, Riley J, Zhang S-D (2010). Doxorubicin in vivo rapidly alters expression and translation of myocardial electron transport chain genes, leads to ATP loss and caspase 3 activation. PLoS ONE.

[37] Ichikawa Y, Ghanefar M, Bayeva M, Wu R, Khechaduri A, Prasad SVN (2014). Cardiotoxicity of doxorubicin is mediated through mitochondrial iron accumulation. J Clin Invest.

[38] Cascales A, Sánchez-Vega B, Navarro N, Pastor-Quirante F, Corral J, Vicente V (2012). Clinical and genetic determinants of anthracycline-induced cardiac iron accumulation. Int J Cardiol.

[39] Khiati S, Dalla Rosa I, Sourbier C, Ma X, Rao VA, Neckers LM (2014). Mitochondrial topoisomerase I (top1mt) is a novel limiting factor of doxorubicin cardiotoxicity. Clin Cancer Res.

[40] Oikonomou E, Tousoulis D, Siasos G, Zaromitidou M, Papavassiliou AG, Stefanadis C (2011). The role of inflammation in heart failure: new therapeutic approaches. Hellenic J Cardiol.

[41] Pecoraro M, Del Pizzo M, Marzocco S, Sorrentino R, Ciccarelli M, Iaccarino G (2016). Inflammatory mediators in a short-time mouse model of doxorubicin-induced cardiotoxicity. Toxicol Appl Pharmacol.

[42] Thandavarayan RA, Giridharan VV, Arumugam S, Suzuki K, Ko KM, Krishnamurthy P (2015). Schisandrin B prevents doxorubicin induced cardiac dysfunction by modulation of DNA damage, oxidative stress and inflammation through inhibition of MAPK/p53 signaling. PLoS ONE.

[43] Zhu H, Sarkar S, Scott L, Danelisen I, Trush MA, Jia Z (2016). Doxorubicin redox biology: redox cycling, topoisomerase inhibition, and oxidative stress. React Oxyg Species (Apex, NC).

[44] Zhang S, Liu X, Bawa-Khalfe T, Lu L-S, Lyu YL, Liu LF (2012). Identification of the molecular basis of doxorubicin-induced cardiotoxicity. Nat Med.

[45] Jirkovský E, Jirkovská A, Bavlovič-Piskáčková H, Skalická V, Pokorná Z, Karabanovich G (2021). Clinically translatable prevention of anthracycline cardiotoxicity by dexrazoxane is mediated by topoisomerase II beta and not metal chelation. Circ Heart Fail.

[46] Lyu YL, Kerrigan JE, Lin C-P, Azarova AM, Tsai Y-C, Ban Y (2007). Topoisomerase IIβ–mediated DNA double-strand breaks: implications in doxorubicin cardiotoxicity and prevention by dexrazoxane. Cancer Res.

[47] Wang X, Robbins J (2014). Proteasomal and lysosomal protein degradation and heart disease. J Mol Cell Cardiol.

[48] Ranek MJ, Wang X (2009). Activation of the ubiquitin-proteasome system in doxorubicin cardiomyopathy. Curr Hypertens Rep.

[49] Willis MS, Parry TL, Brown DI, Mota RI, Huang W, Beak JY (2019). Doxorubicin exposure causes subacute cardiac atrophy dependent on the striated muscle–specific ubiquitin ligase MuRF1. Circ Heart Fail.

[50] Yamamoto Y, Hoshino Y, Ito T, Nariai T, Mohri T, Obana M (2008). Atrogin-1 ubiquitin ligase is upregulated by doxorubicin via p38-MAP kinase in cardiac myocytes. Cardiovasc Res.

[51] Christidi E, Brunham LR (2021). Regulated cell death pathways in doxorubicin-induced cardiotoxicity. Cell Death Dis.

[52] Li DL, Wang ZV, Ding G, Tan W, Luo X, Criollo A (2016). Doxorubicin blocks cardiomyocyte autophagic flux by inhibiting lysosome acidification. Circulation.

[53] Chen D, Yu W, Zhong C, Hong Q, Huang G, Que D (2022). Elabela ameliorates doxorubicin-induced cardiotoxicity by promoting autophagic flux through TFEB pathway. Pharmacol Res.

[54] Carvalho RA, Sousa RP, Cadete VJ, Lopaschuk GD, Palmeira CM, Bjork JA (2010). Metabolic remodeling associated with subchronic doxorubicin cardiomyopathy. Toxicology.

[55] Hrelia S, Fiorentini D, Maraldi T, Angeloni C, Bordoni A, Biagi PL (2002). Doxorubicin induces early lipid peroxidation associated with changes in glucose transport in cultured cardiomyocytes. Biochim Biophys Acta Biomembr.

[56] Carvalho FS, Burgeiro A, Garcia R, Moreno AJ, Carvalho RA, Oliveira PJ (2014). Doxorubicin-induced cardiotoxicity: from bioenergetic failure and cell death to cardiomyopathy. Med Res Rev.

[57] Russo M, Della Sala A, Tocchetti CG, Porporato PE, Ghigo A (2021). Metabolic aspects of anthracycline cardiotoxicity. Curr Treatment Options Oncol.

[58] Minotti G, Ronchi R, Salvatorelli E, Menna P, Cairo G (2001). Doxorubicin irreversibly inactivates iron regulatory proteins 1 and 2 in cardiomyocytes: evidence for distinct metabolic pathways and implications for iron-mediated cardiotoxicity of antitumor therapy. Cancer Res.

[59] Kotamraju S, Chitambar CR, Kalivendi SV, Joseph J, Kalyanaraman B (2002). Transferrin receptor-dependent iron uptake is responsible for doxorubicin-mediated apoptosis in endothelial cells: role of oxidant-induced iron signaling in apoptosis. J Biol Chem.

[60] Thomas CE, Aust SD (1986). Release of iron from ferritin by cardiotoxic anthracycline antibiotics. Arch Biochem Biophys.

[61] Zhang Y-W, Shi J, Li Y-J, Wei L (2009). Cardiomyocyte death in doxorubicin-induced cardiotoxicity. Arch Immunol Ther Exp.

[62] Leri A, Hosoda T, Rota M, Kajstura J, Anversa P (2007). Myocardial regeneration by exogenous and endogenous progenitor cells. Drug Discov Today Dis Mech.

[63] Piegari E, De Angelis A, Cappetta D, Russo R, Esposito G, Costantino S (2013). Doxorubicin induces senescence and impairs function of human cardiac progenitor cells. Basic Res Cardiol.

[64] Cappetta D, De Angelis A, Sapio L, Prezioso L, Illiano M, Quaini F (2017). Oxidative stress and cellular response to doxorubicin: a common factor in the complex milieu of anthracycline cardiotoxicity. Oxid Med Cell Longev.

[65] Meiners B, Shenoy C, Zordoky BN (2018). Clinical and preclinical evidence of sex-related differences in anthracycline-induced cardiotoxicity. Biol Sex Differ.

[66] Thavendiranathan P, Poulin F, Lim K-D, Plana JC, Woo A, Marwick TH (2014). Use of myocardial strain imaging by echocardiography for the early detection of cardiotoxicity in patients during and after cancer chemotherapy: a systematic review. J Am Coll Cardiol.

[67] Riddell E, Lenihan D (2018). The role of cardiac biomarkers in cardio-oncology. Curr Probl Cancer.

[68] Rüger AM, Schneeweiss A, Seiler S, Tesch H, van Mackelenbergh M, Marmé F (2020). Cardiotoxicity and cardiovascular biomarkers in patients with breast cancer: data from the GeparOcto-GBG 84 trial. J Am Heart Assoc.

[69] De Iuliis F, Salerno G, Taglieri L, De Biase L, Lanza R, Cardelli P (2016). Serum biomarkers evaluation to predict chemotherapy-induced cardiotoxicity in breast cancer patients. Tumor Biol.

[70] Ky B, Putt M, Sawaya H, French B, Januzzi JL, Sebag IA (2014). Early increases in multiple biomarkers predict subsequent cardiotoxicity in patients with breast cancer treated with doxorubicin, taxanes, and trastuzumab. J Am Coll Cardiol.

[71] Bali S, Utaal MS (2019). Serum lipids and lipoproteins: a brief review of the composition, transport and physiological functions. Int J Sci Reports.

[72] Wilson P, Abbott RD, Castelli WP (1988). High density lipoprotein cholesterol and mortality. The Framingham Heart Study. Arteriosclerosis.

[73] Lincoff AM, Nicholls SJ, Riesmeyer JS, Barter PJ, Brewer HB, Fox KAA (2017). Evacetrapib and Cardiovascular Outcomes in High-Risk Vascular Disease. N Engl J Med.

[74] Schwartz GG, Olsson AG, Abt M, Ballantyne CM, Barter PJ, Brumm J (2012). Effects of dalcetrapib in patients with a recent acute coronary syndrome. N Engl J Med.

[75] Bowman L, Hopewell JC, Chen F, Wallendszus K, Stevens W, Group HTRC (2017). Effects of Anacetrapib in Patients with Atherosclerotic Vascular Disease. N Engl J Med.

[76] Barter PJ, Kastelein JJ (2006). Targeting cholesteryl ester transfer protein for the prevention and management of cardiovascular disease. J Am Coll Cardiol.

[77] Rached FH, Chapman MJ, Kontush A (2015). HDL particle subpopulations: Focus on biological function. BioFactors.

[78] Feingold KR, Grunfeld C (2000). Introduction to lipids and lipoproteins.

[79] Rader DJ (2002). High-density lipoproteins and atherosclerosis. Am J Cardiol.

[80] Rysz-Gorzynska M, Banach M (2016). Subfractions of high-density lipoprotein (HDL) and dysfunctional HDL in chronic kidney disease patients. Arch Med Sci.

[81] Karathanasis SK, Freeman LA, Gordon SM, Remaley AT (2017). The Changing Face of HDL and the Best Way to Measure It. Clin Chem.

[82] Brites F, Martin M, Guillas I, Kontush A (2017). Anti-oxidative activity of high-density lipoprotein (HDL): Mechanistic insights into potential clinical benefit. BBA Clin.

[83] Mackness M, Mackness B (2015). Human paraoxonase-1 (PON1): Gene structure and expression, promiscuous activities and multiple physiological roles. Gene.

[84] Kontush A, Lindahl M, Lhomme M, Calabresi L, Chapman MJ, Davidson WS, von Eckardstein A, Dimitris K (2015). Structure of HDL: particle subclasses and molecular components. High Density Lipoproteins.

[85] Mackness MI, Arrol S, Abbott C, Durrington PN (1993). Protection of low-density lipoprotein against oxidative modification by high-density lipoprotein associated paraoxonase. Atherosclerosis.

[86] Chistiakov DA, Melnichenko AA, Orekhov AN, Bobryshev YV (2017). Paraoxonase and atherosclerosis-related cardiovascular diseases. Biochimie.

[87] Stafforini DM (2009). Biology of platelet-activating factor acetylhydrolase (PAF-AH, lipoprotein associated phospholipase A 2). Cardiovasc Drugs Ther.

[88] Arakawa H, Qian J-Y, Baatar D, Karasawa K, Asada Y, Sasaguri Y (2005). Local expression of platelet-activating factor-acetylhydrolase reduces accumulation of oxidized lipoproteins and inhibits inflammation, shear stress-induced thrombosis, and neointima formation in balloon-injured carotid arteries in nonhyperlipidemic rabbits. Circulation.

[89] Theilmeier G, De Geest B, Van Veldhoven PP, Stengel D, Michiels C, Lox M (2000). HDL associated PAF-AH reduces endothelial adhesiveness in apoE−/− mice. FASEB J.

[90] Brulhart-Meynet MC, Braunersreuther V, Brinck J, Montecucco F, Prost JC, Thomas A (2015). Improving reconstituted HDL composition for efficient post-ischemic reduction of ischemia reperfusion injury. PLoS ONE.

[91] Ke M, Tang Q, Pan Z, Yin Y, Zhang L, Wen K (2019). Sphingosine-1-phosphate attenuates hypoxia/reoxygenation-induced cardiomyocyte injury via a mitochondrial pathway. Biochem Biophys Res Commun.

[92] Frias MA, Lecour S, James RW, Pedretti S (2012). High density lipoprotein/sphingosine-1-phosphate-induced cardioprotection: Role of STAT3 as part of the SAFE pathway. JAKSTAT.

[93] Kelly-Laubscher RF, King JC, Hacking D, Somers S, Hastie S, Stewart T (2014). Cardiac pre conditioning with sphingosine-1-phosphate requires activation of signal transducer and activator of transcription-3. Cardiovasc J Afr.

[94] Rizzo M, Otvos J, Nikolic D, Montalto G, Toth P, Banach M (2014). Subfractions and subpopulations of HDL: an update. Curr Med Chem.

[95] Yoshikawa M, Sakuma N, Hibino T, Sato T, Fujinami T (1997). HDL3 exerts more powerful anti-oxidative, protective effects against copper-catalyzed LDL oxidation than HDL2. Clin Biochem.

[96] Ashby D, Gamble J, Vadas M, Rye K-A, Barter P (1997). Inhibition of endothelial cell adhesion molecule expression by high density lipoprotein subfractions. Atherosclerosis.

[97] Camont L, Lhomme M, Rached F, Le Goff W, Nègre-Salvayre A, Salvayre R (2013). Small, dense high-density lipoprotein-3 particles are enriched in negatively charged phospholipids: relevance to cellular cholesterol efflux, anti-oxidative, antithrombotic, anti-inflammatory, and antiapoptotic functionalities. Arterioscler Thromb Vasc Biol.

[98] Camont L, Chapman J, Kontush A (2011). Functionality of HDL particles: Heterogeneity and relationships to cardiovascular disease. Arch Cardiovasc Dis Suppl.

[99] Beazer JD, Patanapirunhakit P, Gill JM, Graham D, Karlsson H, Ljunggren S (2020). High-density lipoprotein’s vascular protective functions in metabolic and cardiovascular disease–could extracellular vesicles be at play?. Clin Sci.

[100] de Boer C, Calder B, Blackhurst D, Marais D, Blackburn J, Steinmaurer M (2021). Analysis of the regenerative capacity of human serum exosomes after a simple multistep separation from lipoproteins. J Tissue Eng Regen Med.

[101] Freedman DS, Otvos JD, Jeyarajah EJ, Shalaurova I, Cupples LA, Parise H (2004). Sex and age differences in lipoprotein subclasses measured by nuclear magnetic resonance spectroscopy: the Framingham Study. Clin Chem.

[102] El Khoudary SR, Chen X, Nasr AN, Billheimer J, Brooks MM, McConnell D (2021). HDL (High-Density Lipoprotein) Subclasses, Lipid Content, and Function Trajectories Across the Menopause Transition: SWAN-HDL Study. Arterioscler Thromb Vasc Biol.

[103] Hunter WG, McGarrah RW, Kelly JP, Khouri MG, Craig DM, Haynes C (2019). High-density lipoprotein particle subfractions in heart failure with preserved or reduced ejection fraction. J Am Coll Cardiol.

[104] Soares AAS, Carvalho LSF, Bonilha I, Virginio VW, Nadruz Junior W, Coelho-Filho OR (2019). Adverse interaction between HDL and the mass of myocardial infarction. Atherosclerosis.

[105] Navab M, Hama SY, Hough GP, Subbanagounder G, Reddy ST, Fogelman AM (2001). A cell-free assay for detecting HDL that is dysfunctional in preventing the formation of or inactivating oxidized phospholipids. J Lipid Res.

[106] Weichhart T, Kopecky C, Kubicek M, Haidinger M, Doller D, Katholnig K (2012). Serum amyloid A in uremic HDL promotes inflammation. J Am Soc Nephrol.

[107] Generoso G, Bensenor IM, Santos RD, Staniak HL, Sharovsky R, Santos IS (2019). High-density Lipoprotein-cholesterol Subfractions and Coronary Artery Calcium: The ELSA-Brasil Study. Arch Med Res.

[108] Hoang A, Murphy A, Coughlan M, Thomas M, Forbes J, O’brien R (2007). Advanced glycation of apolipoprotein AI impairs its anti-atherogenic properties. Diabetologia.

[109] McEneny J, Blair S, Woodside JV, Murray L, Boreham C, Young IS (2013). High-density lipoprotein subfractions display proatherogenic properties in overweight and obese children. Pediatr Res.

[110] Zhang Y, Li S, Xu R-X, Guo Y-L, Wu N-Q, Zhu C-G (2015). Distribution of high-density lipoprotein subfractions and hypertensive status: A cross-sectional study. Medicine.

[111] Morgantini C, Natali A, Boldrini B, Imaizumi S, Navab M, Fogelman AM (2011). Anti-inflammatory and antioxidant properties of HDLs are impaired in type 2 diabetes. Diabetes.

[112] Barter P (2002). Effects of inflammation on high-density lipoproteins. Arterioscler Thromb Vasc Biol.

[113] Denimal D, de Barros J-PP, Petit J-M, Bouillet B, Verges B, Duvillard L (2015). Significant abnormalities of the HDL phosphosphingolipidome in type 1 diabetes despite normal HDL cholesterol concentration. Atherosclerosis..

[114] Chan GK, Witkowski A, Gantz DL, Zhang TO, Zanni MT, Jayaraman S (2015). Myeloperoxidase-mediated methionine oxidation promotes an amyloidogenic outcome for apolipoprotein AI. J Biol Chem.

[115] Gugliucci A, Stahl A (1991). In vitro glycation of human apolipoprotein AI reduces its efficiency in lecithin: cholesterol acyltransferase activation. Clin Chim Acta.

[116] Nobecourt E, Davies M, Brown B, Curtiss L, Bonnet D, Charlton F (2007). The impact of glycation on apolipoprotein AI structure and its ability to activate lecithin: cholesterol acyltransferase. Diabetologia.

[117] Phillips MC (2013). New insights into the determination of HDL structure by apolipoproteins1: Thematic Review Series: High Density Lipoprotein Structure, Function, and Metabolism. J Lipid Res.

[118] DiDonato JA, Huang Y, Aulak KS, Even-Or O, Gerstenecker G, Gogonea V (2013). Function and distribution of apolipoprotein A1 in the artery wall are markedly distinct from those in plasma. Circulation.

[119] Shao B, Tang C, Heinecke JW, Oram JF (2010). Oxidation of apolipoprotein AI by myeloperoxidase impairs the initial interactions with ABCA1 required for signaling and cholesterol export 1. J Lipid Res.

[120] Bekhet OH, Zeljkovic A, Vekic J, Paripovic D, Janac J, Joksic J (2016). Hypertension, lipoprotein subclasses and lipid transfer proteins in obese children and adolescents. Scand J Clin Lab Invest.

[121] Woudberg NJ, Goedecke JH, Blackhurst D, Frias M, James R, Opie LH (2016). Association between ethnicity and obesity with high-density lipoprotein (HDL) function and subclass distribution. Lipids Health Dis.

[122] Slagter SN, van Vliet-Ostaptchouk JV, Vonk JM, Boezen HM, Dullaart RP, Kobold ACM (2013). Associations between smoking, components of metabolic syndrome and lipoprotein particle size. BMC Med.

[123] Brinck JW, Thomas A, Brulhart-Meynet MC, Lauer E, Frej C, Dahlback B (2018). High-density lipoprotein from end-stage renal disease patients exhibits superior cardioprotection and increase in sphingosine-1-phosphate. Eur J Clin Invest.

[124] Lappegård KT, Kjellmo CA, Hovland A (2021). High-Density Lipoprotein Subfractions: Much Ado about Nothing or Clinically Important?. Biomedicines.

[125] Munir R, Usman H, Hasnain S, Smans K, Kalbacher H, Zaidi N (2014). Atypical plasma lipid profile in cancer patients: cause or consequence?. Biochimie.

[126] Danilo C, Frank PG (2012). Cholesterol and breast cancer development. Curr Opin Pharmacol.

[127] Abdelsalam KEA, Hassan IK, Sadig IA (2012). The role of developing breast cancer in alteration of serum lipid profile. J Res Med Sci.

[128] Shah FD, Shukla SN, Shah PM, Patel HR, Patel PS (2008). Significance of alterations in plasma lipid profile levels in breast cancer. Integr Cancer Ther.

[129] Williams PT, Vranizan KM, Austin MA, Krauss RM (1993). Associations of age, adiposity, alcohol intake, menstrual status, and estrogen therapy with high-density lipoprotein subclasses. Arterioscler Thromb.

[130] Woudberg NJ, Mendham AE, Katz AA, Goedecke JH, Lecour S (2018). Exercise intervention alters HDL subclass distribution and function in obese women. Lipids Health Dis.

[131] Michalaki V, Koutroulis G, Syrigos K, Piperi C, Kalofoutis A (2005). Evaluation of serum lipids and high-density lipoprotein subfractions (HDL2, HDL3) in postmenopausal patients with breast cancer. Mol Cell Biochem.

[132] Schreier LE, Berg GA, Basilio FM, Lopez GI, Etkin AE, Wikinski RL (1999). Lipoprotein alterations, abdominal fat distribution and breast cancer. IUBMB Life.

[133] Flote VG, Vettukattil R, Bathen TF, Egeland T, McTiernan A, Frydenberg H (2016). Lipoprotein subfractions by nuclear magnetic resonance are associated with tumor characteristics in breast cancer. Lipids Health Dis.

[134] Bani I, Williams CM, Boulter P, Dickerson J (1986). Plasma lipids and prolactin in patients with breast cancer. Br J Cancer.

[135] Knapp ML, Al-Sheibani S, Riches PG (1991). Alterations of serum lipids in breast cancer: effects of disease activity, treatment, and hormonal factors. Clin Chem.

[136] Agurs-Collins T, Kim KS, Dunston GM, Adams-Campbell LL (1998). Plasma lipid alterations in African-American women with breast cancer. J Canc Res Clinical Oncol.

[137] Han C, Zhang H-T, Du L, Liu X, Jing J, Zhao X (2005). Serum levels of leptin, insulin, and lipids in relation to breast cancer in china. Endocrine.

[138] Hasija K, Bagga HK (2005). Alterations of serum cholesterol and serum lipoprotein in breast cancer of women. Indian J Clin Biochem.

[139] Delimaris I, Faviou E, Antonakos G, Stathopoulou E, Zachari A, Dionyssiou-Asteriou A (2007). Oxidized LDL, serum oxidizability and serum lipid levels in patients with breast or ovarian cancer. Clin Biochem.

[140] Owiredu W, Donkor S, Addai BW, Amidu N (2009). Serum lipid profile of breast cancer patients. Pak J Biol Sci.

[141] Bhat SA, Mir MR, Majid S, Reshi AA, Husain I, Hassan T (2013). Serum lipid profile of breast cancer patients in Kashmir. A J Physiol Biochem Pharmacol.

[142] Laisupasin P, Thompat W, Sukarayodhin S, Sornprom A, Sudjaroen Y (2013). Comparison of Serum Lipid Profiles between Normal Controls and Breast Cancer Patients. J Lab Physicians.

[143] Akalanka HMK, Ekanayake S, Samarasinghe K (2018). Could Anthropometric and Lipid Parameters Reflect Susceptibility to Breast Cancer? Comparison of Newly Diagnosed Breast Cancer and Apparently Healthy Women. Asian Pac J Cancer Prev.

[144] Li X, Liu Z-l, Wu Y-t, Wu H, Dai W, Arshad B (2018). Status of lipid and lipoprotein in female breast cancer patients at initial diagnosis and during chemotherapy. Lipids Health Dis.

[145] Pirro M, Ricciuti B, Rader DJ, Catapano AL, Sahebkar A, Banach M (2018). High density lipoprotein cholesterol and cancer: Marker or causative?. Prog Lipid Res.

[146] Ganjali S, Ricciuti B, Pirro M, Butler AE, Atkin SL, Banach M (2019). High-Density Lipoprotein Components and Functionality in Cancer: State-of-the-Art. Trends Endocrinol Metab.

[147] Arenas M, García-Heredia A, Cabré N, Luciano-Mateo F, Hernández-Aguilera A, Sabater S (2017). Effect of radiotherapy on activity and concentration of serum paraoxonase-1 in breast cancer patients. PLoS ONE.

[148] Özmen HK, Askın S (2013). Lecithin: cholesterol acyltransferase and Na+-K+-ATPase activity in patients with breast cancer. J Breast Cancer.

[149] Marinho AT, Lu H, Pereira SA, Monteiro E, Gabra H, Recchi C (2019). Anti-tumorigenic and platinum-sensitizing effects of apolipoprotein A1 and apolipoprotein A1 mimetic peptides in ovarian cancer. Front Pharmacol.

[150] Zamanian-Daryoush M, Lindner D, Tallant TC, Wang Z, Buffa J, Klipfell E (2013). The Cardioprotective Protein Apolipoprotein A1 Promotes Potent Anti-tumorigenic Effects. J Biol Chem.

[151] Tian W, Yao Y, Fan G, Zhou Y, Wu M, Xu D (2019). Changes in lipid profiles during and after (neo) adjuvant chemotherapy in women with early-stage breast cancer: A retrospective study. PLoS ONE.

[152] Sharma M, Tuaine J, McLaren B, Waters DL, Black K, Jones LM (2016). Chemotherapy Agents Alter Plasma Lipids in Breast Cancer Patients and Show Differential Effects on Lipid Metabolism Genes in Liver Cells. PLoS ONE.

[153] Godinho-Mota JCM, Mota JF, Gonçalves LV, Soares LR, Schincaglia RM, Prado CM (2021). Chemotherapy negatively impacts body composition, physical function and metabolic profile in patients with breast cancer. Clin Nutr.

[154] Qi A, Li Y, Yan S, Sun H, Chen Y (2021). Effect of anthracycline-based postoperative chemotherapy on blood glucose and lipid profiles in patients with invasive breast cancer. Ann Palliat Med.

[155] Cuchel M, Rader DJ (2006). Macrophage reverse cholesterol transport: key to the regression of atherosclerosis?. Circulation.

[156] Frias MA, James RW, Gerber-Wicht C, Lang U (2009). Native and reconstituted HDL activate Stat3 in ventricular cardiomyocytes via ERK1/2: role of sphingosine-1-phosphate. Cardiovasc Res.

[157] Durham KK, Chathely KM, Mak KC, Momen A, Thomas CT, Zhao Y-Y (2018). HDL protects against doxorubicin-induced cardiotoxicity in a scavenger receptor class B type 1-, PI3K-, and Akt-dependent manner. Am J Physiol Heart Circ Physiol.

[158] Durham KK, Kluck G, Mak KC, Deng YD, Trigatti BL (2019). Treatment with apolipoprotein A1 protects mice against doxorubicin-induced cardiotoxicity in a scavenger receptor class B, type I-dependent manner. Am J Physiol Heart Circ Physiol.

[159] Wojtacki J, Lewicka-Nowak E, Lesniewski-Kmak K (2000). Anthracycline-induced cardiotoxicity: clinical course, risk factors, pathogenesis, detection and prevention-review of the literature. Med Sci Monit.

[160] Solomon R, Gabizon AA (2008). Clinical pharmacology of liposomal anthracyclines: focus on pegylated liposomal doxorubicin. Clin Lymphoma Myeloma Leuk.

[161] Wang B, Yuan Y, Han L, Ye L, Shi X, Feng M (2014). Recombinant lipoproteins reinforce cytotoxicity of doxorubicin to hepatocellular carcinoma. J Drug Target.

[162] Yuan Y, Wang W, Wang B, Zhu H, Zhang B, Feng M (2013). Delivery of hydrophilic drug doxorubicin hydrochloride-targeted liver using apoAI as carrier. J Drug Target.

[163] Jaouad L, de Guise C, Berrougui H, Cloutier M, Isabelle M, Fulop T (2006). Age-related decrease in high-density lipoproteins antioxidant activity is due to an alteration in the PON1’s free sulfhydyl groups. Atherosclerosis.

[164] Khalil A, Jay-Gerin J-P, Fülöp T (1998). Age-related increased susceptibility of high-density lipoproteins (HDL) to in vitro oxidation induced by γ-radiolysis of water. FEBS lett.

[165] Lyu L-C, Yeh C-Y, Lichtenstein AH, Li Z, Ordovas JM, Schaefer EJ (2001). Association of sex, adiposity, and diet with HDL subclasses in middle-aged Chinese. Am J Clin Nutr.

